# Non-invasive authentication of mail packages using nuclear quadrupole resonance spectroscopy

**DOI:** 10.1038/s41598-023-31497-9

**Published:** 2023-04-04

**Authors:** Kelsey Horace-Herron, Naren Vikram Raj Masna, Peyman Dehghanzadeh, Soumyajit Mandal, Swarup Bhunia

**Affiliations:** 1 Department of Electrical and Computer Engineering University of Florida, Gainesville, FL 32611 USA; 2 Instrumentation Division Brookhaven National Laboratory Upton, New York, 11973 USA

**Keywords:** Engineering, Electrical and electronic engineering

## Abstract

The international postal network is one of the most widely used methods for correspondence throughout the world. Most postal traffic across the globe consists of legitimate interpersonal, business-consumer, and business-business communications. However, the global postal system is also utilized for criminal activity. In particular, it is often utilized to ship and distribute contraband, including illegal psychoactive drugs such as fentanyl and heroin, to consumers. Existing technological solutions are capable of identifying synthetic opioids and other illegal drugs within packages, but are accompanied by several disadvantages that make them unsuitable for large-scale authentication of international mail traffic. This paper presents a novel method for non-invasive authentication of mail packages that overcomes these challenges. The approach uses nuclear quadrupole resonance (NQR) spectroscopy to detect and quantify the presence of known active pharmaceutical ingredients (APIs) within the package. It has been experimentally demonstrated using a bench top prototype. Test results from a variety of package types demonstrate the effectiveness of the proposed authentication approach.

## Introduction

Tracing its roots back to the 1700s, the United States Postal Service (USPS) is a governmental agency that has expanded over time, becoming the most reliable mailing company throughout the United States. Because of its exponential growth, consumers have greatly benefited from the rapid advancements and feasibility the postal system has contributed over the years. While USPS has worked diligently to become a household brand and earn consumers’ trust, the lack of an updated security structure for customers’ safety has been questioned. With USPS being the primary source for delivering mail correspondence, drug traffickers have exploited the popular postal service by shipping contraband, illegal substances, and many other hazardous items. Drug companies that distribute illegal substances target the public postal service since USPS is prohibited by law from opening packages without a search warrant, unlike private carriers such as UPS and FedEx. A similar situation prevails in many other countries, making mail security a truly global problem. The advent of private online networks (known as the dark web) has further complicated the situation. Opiates, psychoactive drugs, and various illicit drugs can be brought illegally in a matter of seconds from various drug traffickers active on the dark web. Out of 104 illegal drug websites on the dark web, $92\%$ indicated that they used the local postal service, while on the clear web, $80\%$ of the 20 websites visited guided users on how to ship illicit drugs by using the mailing system^1^.

**Figure 1:**
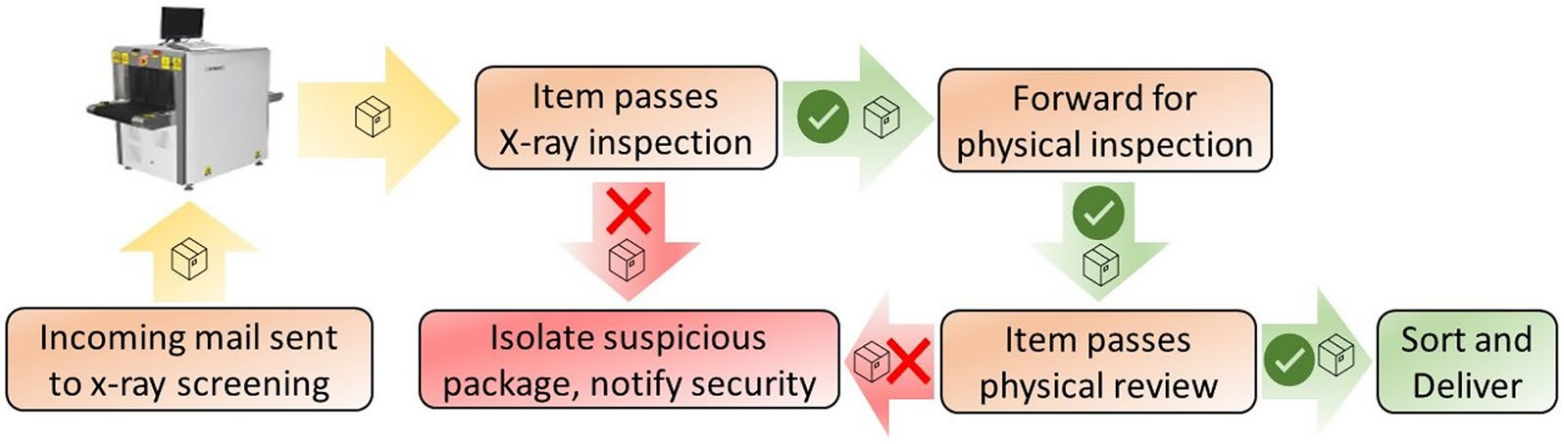
The current screening process followed by most mail services, which does not rely on any quantitative information.

Currently, the most common method of authenticating mail and identifying its contents is restricted to using X-ray scanners. The scan results are then forwarded for physical inspection. Any packages that fail these test are isolated for security. This process is shown in Fig. 1. Over time, drug companies have familiarized themselves with the postal network. They have begun to take advantage of the system by learning to ship packages internationally without an electronic presence. Statistics show that $40\%$ of internationally shipped packages entering the United States are not electronically tracked^2^. This recurring problem is a critical threat to society and in dire need of a solution. Nuclear quadrupole resonance (NQR) spectroscopy has come to the forefront to alleviate several global supply chain security issues. For example, it has already proven effective in authenticating medicines and tracing food products through the supply chain^3–5^. NQR spectroscopy requires minimal sample preparation and is a noninvasive and nondestructive analysis technique, thus making it promising for detecting drugs in opaque packages at any point in the shipment process and eventually reducing the circulation of illegal substances in mail packaging.

**Figure 2:**
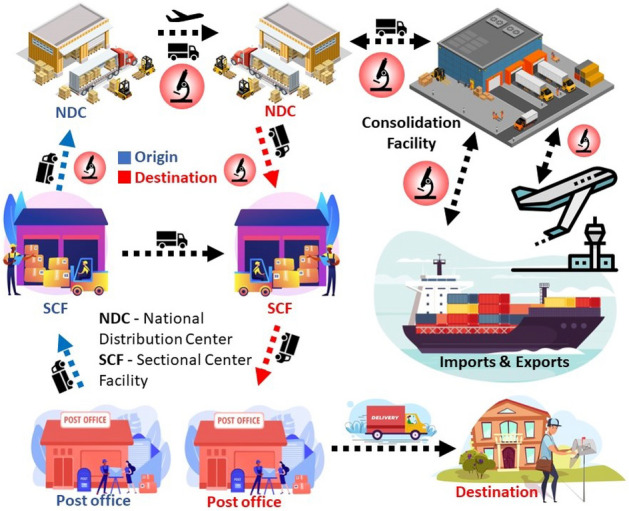
The mail life cycle from origin to destination, which can proceed through different modes^7,8^.

An overview of the logistics involved in a physical mail life cycle is shown in Fig. 2. Here we use the U.S. mail system as a concrete example; other countries have similar life cycles. The U.S. Postal Service (USPS) and most private postal services have standard logistics that willful offenders use to their advantage. When the mail package starts its journey from the origin post office, it goes through a physical screening process, as shown in Fig. 1. There is little to no authentication involved in the later stages. Depending on the destination, the package is sent to a sectional center facility (SCF) for local distribution or from there to the national distribution center (NDC) for interstate deliveries. Most transfers are done by road using trucks except for airmails and express deliveries. A similar situation unfolds in imports and exports, where the sheer volume of the goods does not allow for authentication. Although the packages are tracked during the entire process, their contents are not verified for counterfeit, adulterated, or illegal substances except for physical checking at the origin. Our authentication model can be easily integrated into the mail life cycle while transferring the packages between SCFs, NDCs, consolidation facilities, and import/export terminals.

In our earlier work, we demonstrated that NQR could be used to authenticate drugs and food products in the market. The food manufacturer can measure the spectrum of a food product and upload it to a cloud, allowing quality checks to be performed at various points in the supply chain verify the authenticity of the food item^4^. Here we focus on NQR’s ability to detect drugs through typical mailing packages, which enables consumers to authenticate drugs before the packages are opened and consumed. Drug traffickers are, unknowingly to consumers, increasingly mixing highly potent drugs such as fentanyl with heroin and cocaine, leading to an epidemic of deaths^6^. Just opening a package containing fentanyl can be dangerous, as only $\sim$2 mg is needed to overdose. Permitting consumers to authenticate the drugs they are consuming (whether legal or otherwise) will save lives by giving them power over their choices. This paper proposes a novel authentication model using our current NQR setup and proves its efficiency at detecting drugs in realistic volumes (a few grams) after they are placed in different types of packaging. Manufacturers can also use this technique for quality control of their packages by testing them quantitatively at different stages of the supply chain.

## Literature review and background

NQR has recently been gaining traction as an analytical technique due to multiple reasons: (1) *High specificity*: NQR can be used for an exact determination of the local distribution of electron density in molecules, giving more accurate results than NMR chemical shifts^9^. (2) *Non-invasiveness*: NQR is packaging-insensitive and requires little sample preparation. (3) *Ease of use*: The applicability of NQR is not limited by the need for an external magnetic field, unlike the case with other magnetic resonance techniques^10^. However, NQR also has several drawbacks that researchers focus on addressing, such as relatively low sensitivity^11^ and strong dependence of the measured signal on excitation sequence parameters (such as power level, pulse width, and pulse repetition rate)^12^. Even with these drawbacks, NQR is by far the most economical effective technique, as seen in Table 1. Unlike the alternative methods, which have a hefty price tag, the applications of NQR are not limited to professional/laboratory personnel; instead, they are commercially feasible for average consumers.

**Table 1:** Comparison of different authentication models currently being used for detecting illegal substances.

Num	Application	Price ($)
1	TruNarc	> 30,000
2	X-Ray screening machine	> 10,000
3	Nuclear magnetic resonance (NMR)	> 35,000
4	Infrared spectroscopy (IR)	> 5000
5	Mass spectroscopy	10,000 to 100,000
6	Near infrared (NIR)—desktop unit	10,000 to 12,000
7	NIR—portable unit	16,000
8	NIR—online unit	12000 to 25,000
9	NIR—inline unit	25,000
10	NQR (proposed method)	<3000

While other techniques such as Raman spectroscopy, nuclear magnetic resonance (NMR), and thermal desorption ambient mass spectroscopy are currently used by law enforcement officers in the field to detect drugs, these devices are expensive and suffer from several drawbacks (Table 1). Raman spectrometers, such as those developed by TruNarc, can only scan drugs through translucent materials^13^. Also, drugs cannot be detected when packages have been heat sealed and have yet to be opened^14^. Terahertz (THz) spectroscopy has great potential for sensing a wide range of molecules. However, studies are practically limited to macroscopic ensembles of compounds (e.g., dense pellets of crystallized molecules or highly concentrated solutions of nanomaterials) due to the long radiation wavelength^15^. Lastly, IR spectroscopy can identify chemical substances or functional groups in solid, liquid, or gaseous forms. While IR has many uses, it does not provide information about the relative locations of the functional groups in a molecule. On the other hand, NMR is most suitable for detecting liquid samples, and requires expensive and time-consuming sample preparation for analyzing pill and powder products^16^. By contrast, NQR spectroscopy is a solid-state analytical technique with high resolution and low sensitivity to surface particles, thus allowing it to detect drugs noninvasively through an wide variety of opaque packaging materials with little to no sample preparation.

The active pharmaceutical ingredients (APIs) of most legal and illegal drugs contain NQR-active quadrupolar nuclei such as $^{14}$N and $^{35}$Cl. Thus, numerous publications discuss approaches that utilize NQR spectroscopy to detect illegal and counterfeit substances. In^17^, a team based in the U.K. developed a $^{14}$N NQR spectroscopy system and quantitative analysis procedure for detecting counterfeit samples of the antimalarial drug Metakelfin. The researchers compared a batch of fake Metakelfin samples to a genuine batch obtained from the manufacturer. Metakelfin contains two APIs (sulfalene and pyrimethamine), and the NQR analysis revealed spectral differences for the sulfalene component indicative of differences in the processing history of the two batches. Specifically, the NQR analysis showed that the suspected counterfeit tablets contained only $43 \pm 3\%$ as much sulfalene as the genuine Metakelfin tablets. The team went on to show that Fourier transform (FT)-Raman and FT-near infrared (NIR) spectroscopy only achieved differentiation between the batches but no ascription, thus proving NQR as a means for nondestructive identification and content-indicating first stage analysis of counterfeit pharmaceuticals.

Another group of researchers proposed an NQR system designed to detect prohibited substances in the frequency range of $0.4{-}6$ MHz using low-power and frequency-swept excitation^18^. The team presented their system’s components and several technical solutions for noise reduction during data acquisition, such as using fiber-optic communication and a battery-powered low-noise amplifier (LNA). The system’s performance was evaluated using sodium nitrite and paracetamol; the maximum detection distance for paracetamol was 2 cm and the minimum detection limit for sodium nitrite was 1 g at a transmitted power of 7 W. Performance was further validated by analyzing distance and substance quantity versus power level. Further research is needed to implement solutions to cancel coherent noise and enable analysis of substances with resonance frequencies below 1 MHz.

A research team at Tokyo Customs Lab created an NQR application to detect the illegal psychoactive drug methamphetamine. The team proposed a novel mathematical approach for signal detection and parameter estimation using NQR^19^. They also addressed the issue of detecting methamphetamine in various situations. Experiments were conducted using the novel algorithm (named EPIC) and a solenoid sample coil. The EPIC algorithm efficiently detected the illegal substance in the presence of noise and interference. However, the use of an enclosed detector geometry (i.e., the solenoid coil) prevents the analysis of large samples. Past research has also utilized NQR to detect lethal items such as explosives and landmines. For example, in^20^, the authors proposed a robust algorithm based on singular value decomposition (SVD) to detect NQR signals in explosives. Such algorithms are required since the practical use of NQR is restricted by the inherently low signal-to-noise ratio (SNR) of the observed signals^20^.

**Figure 3:**
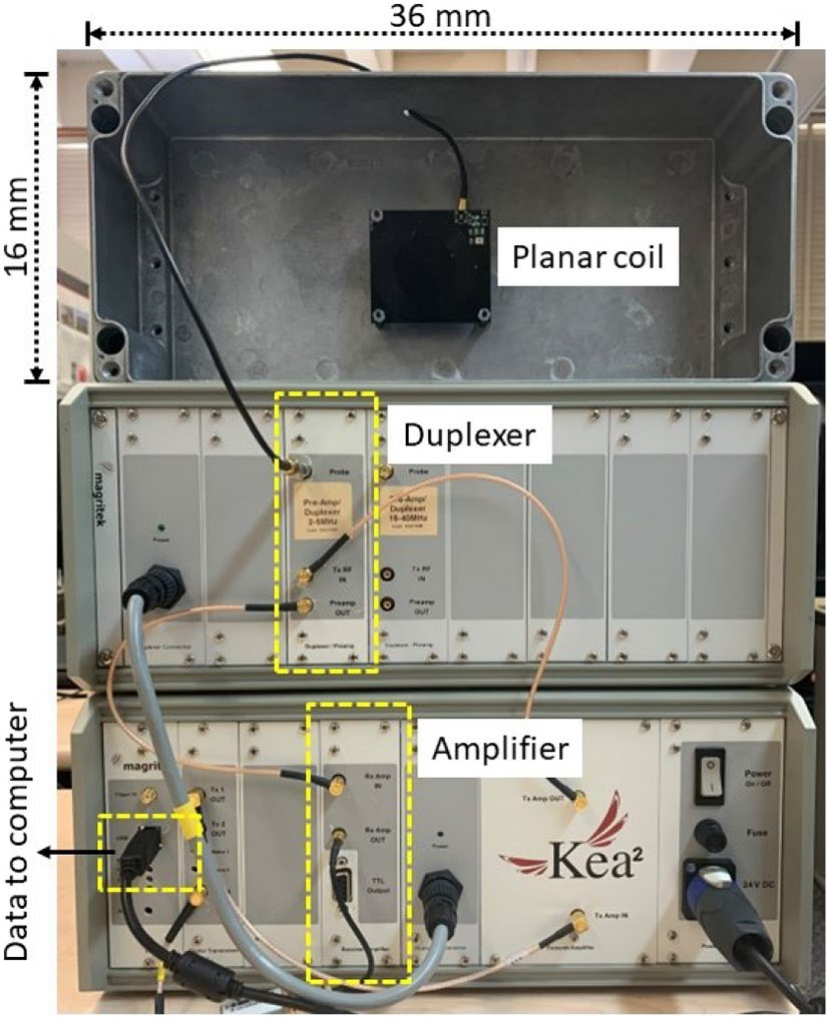
The experimental setup for detecting psychoactive drugs consists of a custom planar coil, a Magritek Kea2 benchtop spectrometer with an internal receiver, and a duplexer. The spectrometer is connected to a computer to collect data.

**Figure 4:**
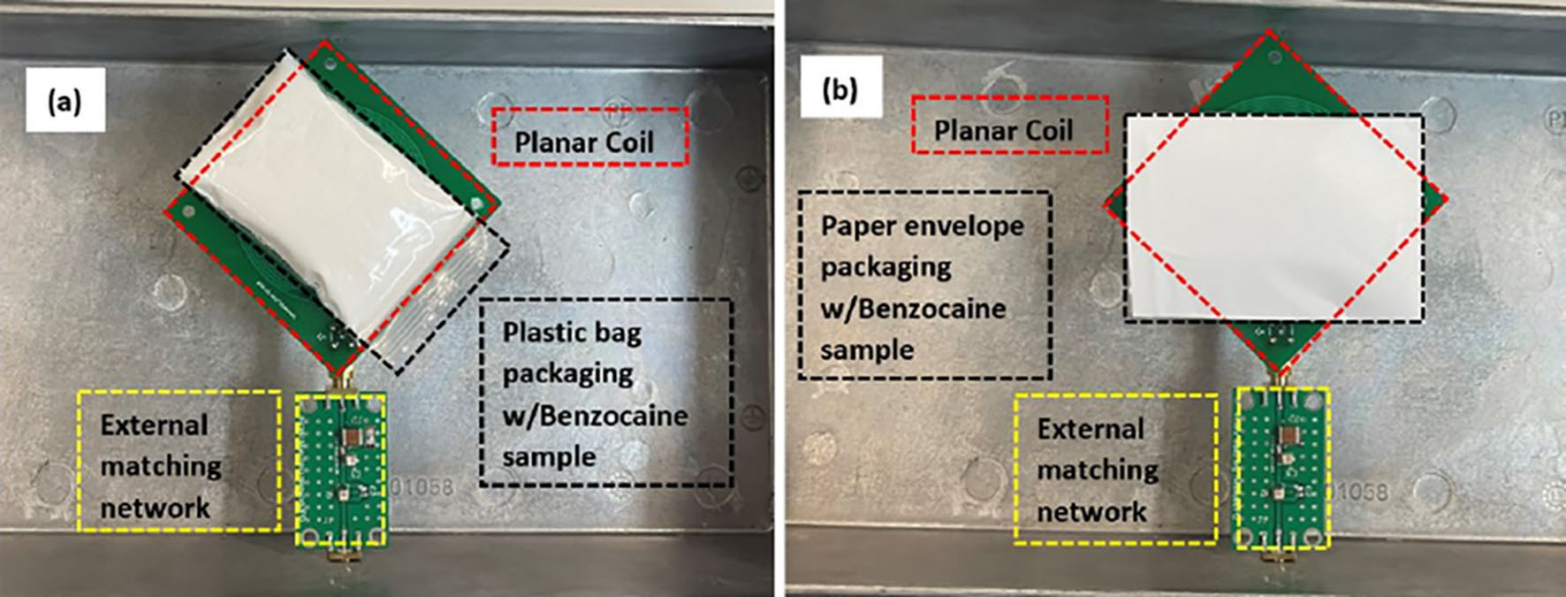
Psychoactive drug (a benzocaine sample) placed in various types of mail packaging for testing using a planar coil with an external matching network: (**a**) plastic bag, and (**b**) paper envelope. All are encased inside a aluminium Faraday cage.

We propose a noninvasive NQR-based approach for detecting illegal substances within mail packages. This paper focuses on detection of benzocaine, a topical analgesic found in different over-the-counter medications used to treat sore throat or relieve mouth pain caused by cold sores, canker sores, and irritation of the mouth and gums. While benzocaine relieves pain, the U.S. Food and Drug Administration (FDA) has issued many warnings surrounding the drug since 2006^21^. Benzocaine has been reported to cause a blood disorder named methemoglobinemia that reduces oxygen supply to tissues and can result in complications such as seizures and heart arrhythmias. A search of the FDA’s Adverse Event Reporting System database through March 16, 2011, identified 21 cases of methemoglobinemia associated with the use of OTC benzocaine gel or liquid products^22^. Here we describe an NQR setup that can detect and quantify benzocaine within different mail packages.

## Theory and system design

NQR spectroscopy is a non-invasive technique to detect APIs in drugs that can be employed without special safety training or protective clothing since it does not emit radiation^3,4^. Its insensitivity to pill coatings and non-metallic packaging material makes it convenient when examining drugs in large quantities. Most medicines have APIs that contain an NQR-active quadrupolar nucleus (spin number $I\ge 1$) such as $^{14}$N or $^{35}$Cl. Approximately $50\%$ of all nuclei in the periodic table are quadrupolar and thus NQR-active.

### NQR spectroscopy for API analysis

NQR spectra are generated by interactions between the electric quadrupole moments of specific nuclei and their local electric field gradient (EFG) tensor. This paper focuses on $^{14}$N since it is quadrupolar (spin $I = 1$) and present in most APIs. The $^{35}$Cl nucleus, which has $I = \frac{3}{2}$, is also of interest. Typical resonance frequencies for $^{14}$N and $^{35}$Cl range from 0.1 to 5 MHz and 20–40 MHz, respectively.

NQR resonance frequencies are highly sensitive to molecular motion and the dynamic properties of the crystal lattice, i.e., drug chemistry. The amplitude and line width identify additional characteristics relating to the API; the amplitude is proportional to the concentration of the active ingredient, and the line width relates to the physical form of the drug (aging, processing, changes in material properties, and impurities). These relationships often allow one to determine the manufacturer of the drug, as it relates to differences in compacting pressures used during the manufacturing process^4^.

**Figure 5:**
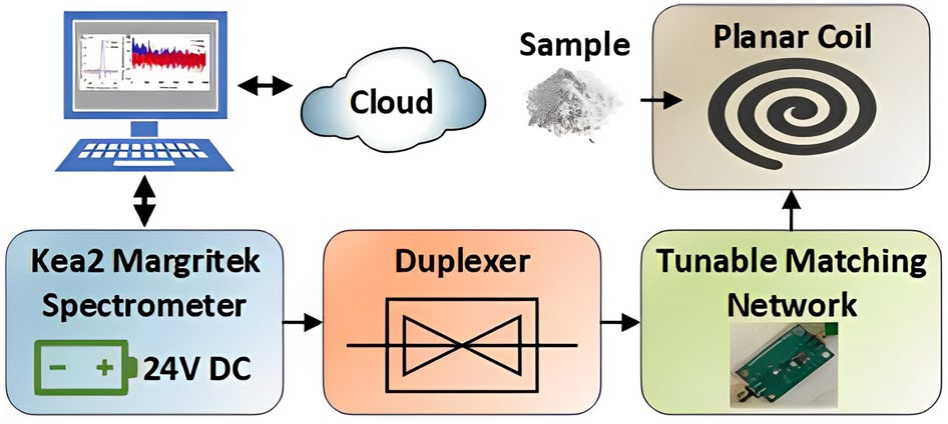
System-level block diagram of mail package authentication using NQR spectroscopy.

### Example API: benzocaine

Benzocaine is an odorless, white, crystalline powder that is commonly used as a topical anesthetic (i.e., to provide comfort and relieve local pain) in a variety of applications ranging from dental procedures to minor traumas. This amino ester is the active ingredient in numerous popular over-the-counter analgesic products such as *Orajel*. Benzocaine is sold in many forms, including sprays, creams, aerosols, and gels, and at various concentrations ranging from $5{-}20\%$. Back in 2018, the U.S. FDA called for manufacturers to place warning labels on over-the-counter (OTC) products such as benzocaine. Because its physical appearance resembles cocaine, dealers have incorporated benzocaine as a cutting agent. As mentioned earlier, it has also been found to be linked to a life-threatening condition named methemoeglobinemia.

Benzocaine molecules reversibly bind to, and inhibit, sodium channels in neuronal cell membranes. When applied, benzocaine enters the cell in non-ionized form, but eventually becomes ionized after traversing the membrane bi-layer. Once ionized, it inhibits voltage-gated sodium channels by binding to their alpha sub-unit. The binding stops cellular depolarization and slows signal conduction^23–26^.

Benzocaine can be purchased in a variety of physical forms. Because our focus is detecting substances in mail packages, we experimented using pure benzocaine powder. The powder was weighed and then packaged evenly in the shipping materials that can be seen in Fig. 13. The packaged mail samples were placed on a planar detector (one at a time) for testing, as described further in the next sub-section.

**Figure 6:**
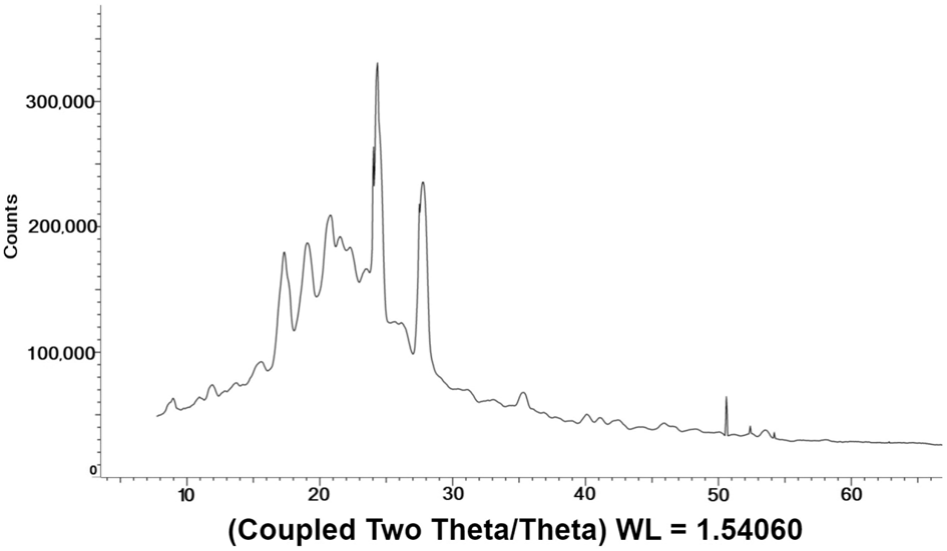
Powder X-ray diffraction (XRD) results for the benzocaine sample.

Since benzocaine is a crystalline compound, it is suitable for detection using NQR spectroscopy. NQR spectra can be used to distinguish between polymorphic crystalline phases of APIs^27^. Benzocaine is known to have three polymorphic forms. However, only two of these forms occur at room temperature: monoclinic (I) and orthorhombic (II). To characterize the polymorph composition of our sample, a powder X-ray diffraction (XRD) experiment was conducted at the University of Florida X-Ray Diffraction Laboratory. The substance was loaded into a capillary tube and mounted onto a Bruker Dual micro source D8 Venture diffractometer equipped with a PHOTON III detector. The data was collected using Cu K-$\alpha$ radiation (8.04 keV, wavelength of 1.54 Å) and integrated using the APEX4 software package. The minimum scan step size that could be reached was $0.02^{\circ }$. The resulting diffraction pattern is shown in Fig. 6. Observed peak broadening is principally due to the 2D detector used in the instrument. After obtaining the diffraction pattern, we searched for the two structural variants on a crystallography database. The corresponding structures were downloaded as CIF files and then used as inputs to free software (VESTA) for predicting the powder XRD patterns. By comparing the predicted and measured peak values, we were able to identify our benzocaine sample as consisting entirely of the orthorhombic (II) polymorph.

### Experimental setup

The experimental setup is shown in Figs. 3 and 4. It consists of an open-geometry detector (a planar coil) to accommodate large samples and an impedance matching network on a printed circuit board (PCB). The planar coil is circular and has outer dimensions of 7 cm $\times$ 7 cm. The windings use copper traces (width $=0.9$ mm, 11 turns, gap $=0.2$ mm). The coil is internally connected to a matching network that is wired to a Magritek Kea2 benchtop spectrometer run off a DC power supply. The spectrometer contains a duplexer to switch between transmit (excitation) and receive (signal detection) modes and an LNA to minimize receiver noise figure (NF). All NQR experiments were controlled from Prospa, a graphical user interface (GUI) developed by Magritek for the Kea2 spectrometer. A Faraday cage (36 cm $\times$ 16 cm $\times$ 9 cm aluminum box) encases the matching network and coil to reduce radio frequency interference (RFI) from the environment. As seen in the system-level block diagram in Figs. 5, 6, the spectrometer is connected to a computer that is connected to the cloud. Cloud connectivity helps access the latest information about the samples and also store private information such as classification models for tracking the origin of the samples.

**Figure 7:**
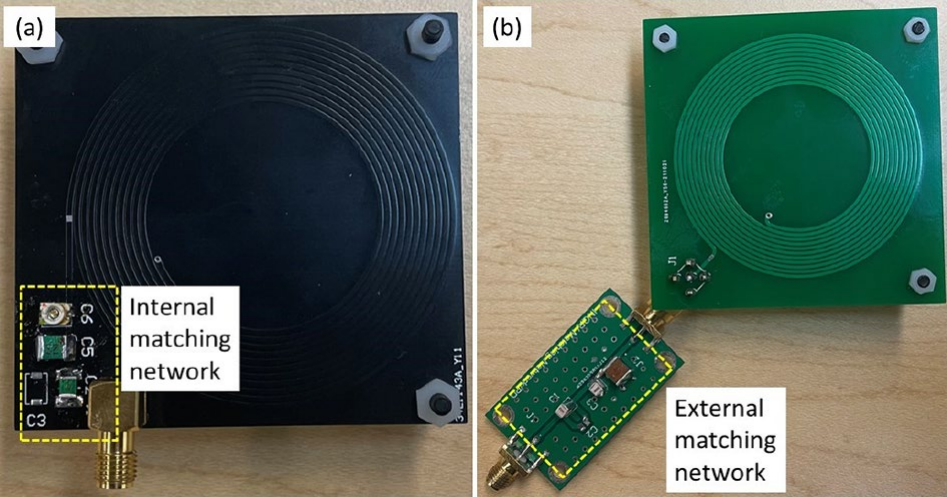
Two versions of the open-geometry detector (a planar coil) were developed: (**a**) with an internal matching network used for fine-grained tuning of the coil; and (**b**) with an option for attaching an external tunable matching network.

Samples were placed on the planar coil, which acts as both transmitter and receiver and is impedance-matched to 50 $\Omega$ near the NQR resonant frequency to maximize power transfer and minimize NF. Tunable series and parallel capacitors in the matching network allow the matching frequency to be adjusted to match that of the sample. We developed two different versions of planar coils (with and without an internal matching network) as shown in Fig. 7. The version shown in Fig. 7a has an internal matching network that is used for fine-grained tuning. Another version, shown in Fig. 7b, has a port to connect to an external matching network. This option increases the usability of the coil for analyzing a variety of samples. The tuned duplexer circuit limits the operating range of the system and delays the onset of self-resonance in the coil. A spin-locked spin echo (SLSE) pulse sequence^28^ was used to measure three important sample-dependent NQR parameters, namely the effective transverse relaxation time constant (denoted by $T_{2,eff}$), spectral linewidth (denoted by $\Delta f$), and initial signal amplitude (denoted by *A*). The SLSE sequence parameters (consisting of the excitation frequency $f_0$, pulse length $t_p$, echo period $t_{E}$, the number of echoes $N_{E}$, and the repetition period $t_{R}$) were optimized to maximize SNR for a given total experiment time^4^.

## Results

This section demonstrates the effectiveness of NQR for non-invasive detection of psychoactive drugs. First, we explain how the proposed coil design helps improve the system’s efficiency. Next, we provide an in-depth description of the test samples utilized for our experiments. Finally, we describe our detection method for the psychoactive drug benzocaine and the effect of different package types on the signal.

### Planar coil properties

There are several advantages to using open-geometry NQR detectors (a planar coil in this case) compared to conventional enclosed-geometry detectors (such as solenoid coils). The main advantage is that it can be used with arbitrary-sized samples, thus allowing a single detector to handle the wide range of mail package sizes encountered in practice (ranging from small envelopes to bulky parcels). In addition, such planar coils can be manufactured with high accuracy and precision using standard PCB fabrication techniques, thus simplifying instrument calibration. By contrast, solenoids are usually hand-wound, which is a time-consuming process with relatively poor precision. On the other hand, enclosed geometries such as solenoids typically have higher detection efficiency. The detection efficiency, $\eta$, of an inductive NQR detector (i.e., coil) is defined as

1 $$\begin{aligned} \eta = \frac{B_1/I_1}{\sqrt{R_s}} \end{aligned}$$

where $B_1/I_1$, also known as the coil sensitivity, is the magnetic field amplitude, $B_1$, generated within the sample volume per unit current, $I_1$, flowing in the coil, and $R_s$ is its series resistance^28^. The measured SNR is increased by averaging the data obtained from $N>1$ SLSE measurements (known as scans); the result is proportional to $\eta ^{2} N$ (in power units). The geometry of the proposed planar coil detector was optimized using electromagnetic (EM) simulations to maximize its detection efficiency.

**Figure 8:**
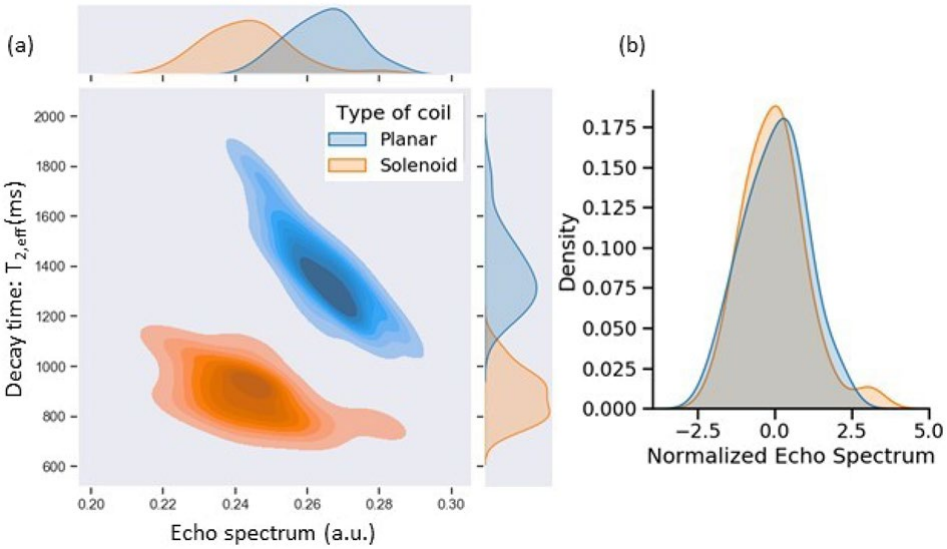
Histogram of NQR signal features measured from multiple experiments on the same sample using two different detector types: planar and solenoid. (**a**) Comparison of the absolute values of initial spectral amplitude, *A*, and effective decay time constant, $T_{2,eff}$, in the 2D plane. (**b**) Comparison of the histograms of *A* obtained from both the coils after normalization.

**Figure 9:**
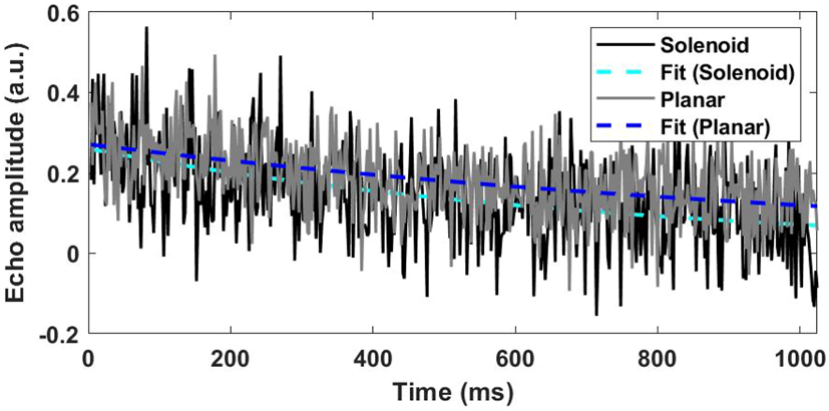
Measured echo decays from an Acetaminophen sample, displaying the contrast in $T_{2,eff}$ values between the solenoid and planar coils.

We validated the proposed open-geometry detector by comparing the NQR signal properties measured for a given sample using the planar coil with those obtained from a reference solenoid coil. In particular, we used both detectors to perform 30 experiments on a given Acetaminophen sample and analyzed the resulting data. The results are summarized in Fig. 8a as a 2D histogram in the $(A, T_{2,eff})$ plane, with darker regions corresponding to a higher probability of obtaining a given combination of *A* and $T_{2,eff}$. The subgraphs on the top and right show the 1D projections of the 2D histogram along the *A* and $T_{2,eff}$ axes, respectively. Both the coils generate Gaussian distributions along the two axes, but with different mean values. The difference between the mean values of *A* measured by the two coils is primarily due to their differing sensitivity functions. This effect has been removed in Fig. 8b by normalizing the *A* axis. The resulting normalized histograms are nearly identical, as expected in the presence of additive white Gaussian noise. However, there is also a significant difference between the mean values of $T_{2,eff}$, as shown in Fig. 9. This quantity is mainly a property of the sample but also depends on the average $B_1$ field amplitude, $\overline{B_{1}}$, present during the SLSE sequence. The latter was different for the two detectors, which explains the results. In fact, $T_{2,eff}$ increases with $\overline{B_{1}}$ for SLSE-like multipulse sequences^29^. Here $\overline{B_{1}}=B_{1}\delta$ where $B_{1}$ is the peak $B_1$ field amplitude during the pulses and $\delta = t_p/t_E$ is the sequence duty cycle (i.e., the fraction of time for which the transmitter is ON).

The effects of $\overline{B_{1}}$ on $T_{2,eff}$ are predictable and can be removed via one-time calibration of the detector for each sample. In addition, we observed small shifts in resonant frequency between the two detectors, likely due to changes in sample temperature. This effect can be removed by measuring the sample temperature and then adjusting the excitation frequency to track the expected shift in NQR resonance frequency.

**Figure 10:**
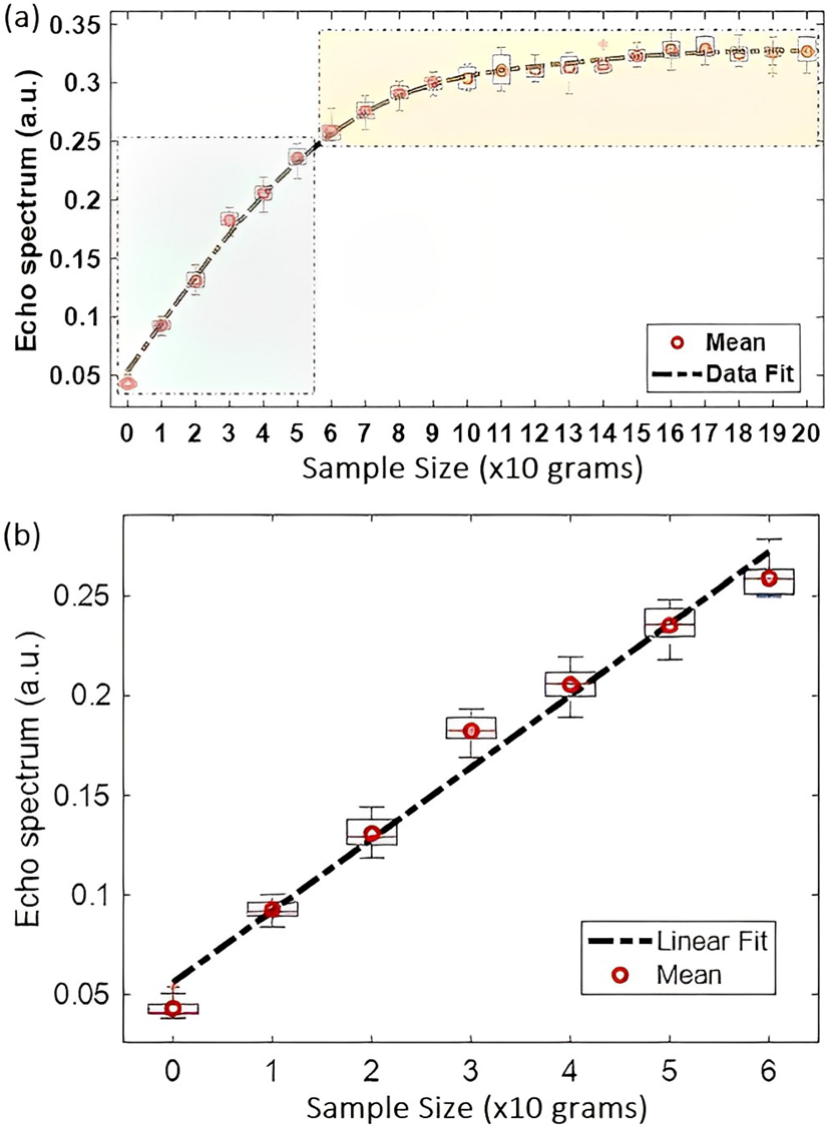
Analysis of planar coil sensitivity. (**a**) The initial amplitude, *A*, of NQR signals measured by the planar coil saturates with increasing sample thickness since the $B_1$ field generated by the planar coil does not uniformly excite all the $^{14}$N nuclei. (**b**) The relationship between *A* and sample thickness is almost linear for thin samples (corresponding to the green region in (**a**)).

Unlike an closed geometry like a solenoid, the strength of the magnetic field, $|B_1|$, generated by an open geometry such as the proposed planar coil is strongly non-uniform and decreases with distance *z* from the coil surface. As a result, the nutation angle of nuclei subject to a pulse of length $t_p$, which is given by $\theta (z) = \gamma B_{1}(z)t_{p}$ where $\gamma$ is the gryomagnetic ratio of the chosen nuclear species, also decreases with depth. The initial signal amplitude, *A*, generated by an SLSE sequence from powder samples containing spin-1 nuclei such as $^{14}$N is known to vary with flip angle as $A\propto J_{3/2}(\theta )$ where $J_{3/2}(\cdot )$ is the Bessel function of order-3/2^29^. This function reaches a maximum at $\theta _{opt}\approx 119.5^{\circ }$, which is known as the optimum flip angle. Due to the strong decrease of $|B_{1}|$ with depth, the distribution of flip angles within the sample also varies with depth, such that most of the signal arises from a thin “slice” around the depth $z_{opt}$ for which $\theta (z)\approx \theta _{opt}$. In addition, due to the principle of reciprocity for EM fields^30^, the signal induced on the coil is proportional to $B_{1}/I_{1}$ and also decreases with depth. Thus, the initial amplitude of the detected signal (which is proportional to the SNR per scan) scales as

2 $$\begin{aligned} A \propto \int _{z_{min}}^{z_{max}}{\left| \frac{B_{1}(z)}{I_{1}}\right| \left( \gamma B_{1}t_{p}\right) dz} \end{aligned}$$

where the sample is assumed to extend over the range $[z_{min},z_{max}]$. Due to the strong decrease of $|B_{1}|$ with depth (approximately as $1/z^{3/2}$), the integral in (2) tends to converge to a maximum value as the thickness of the sample increases. This behavior is visible in the measured data shown in Fig. 10a. The nonlinear relationship between *A* and sample thickness makes it more challenging to reliably estimate the quantity of API in a given sample. However, the relationship is almost linear for thin samples (corresponding to the green region in the figure), as shown in Fig. 10b. Thus, the proposed open-geometry detector is best-suited for quantifying relatively thin samples, such as envelopes.

### Psychoactive drug detection using NQR

As explained in “Literature review and background” section, NQR has been used to detect counterfeit medicines in several studies. However, the intrinsic sensitivity of $^{14}$N NQR is relatively poor due to the low resonant frequencies (1–5 MHz) of most pharmaceutical APIs and the low gyromagnetic ratio of $^{14}$N. The result is low SNR per scan, especially for resonant frequencies $<1$ MHz. Techniques for improving SNR include (1) pre-polarization of $^{14}$N nuclei via adiabatic polarization transfer from nearby protons^31^, and (2) use of alternative detectors, such as SQUIDs^32^, for low-frequency resonances. These SNR enhancement techniques are particularly valuable for small samples with low-frequency resonances ($<1$ MHz). They were not utilized in this study due to (1) the relatively large amounts of API ($>10$ g) within typical packages containing illicit drugs, and (2) the general availability of resonant frequencies in the 2–3 MHz range^17^.

**Figure 11:**
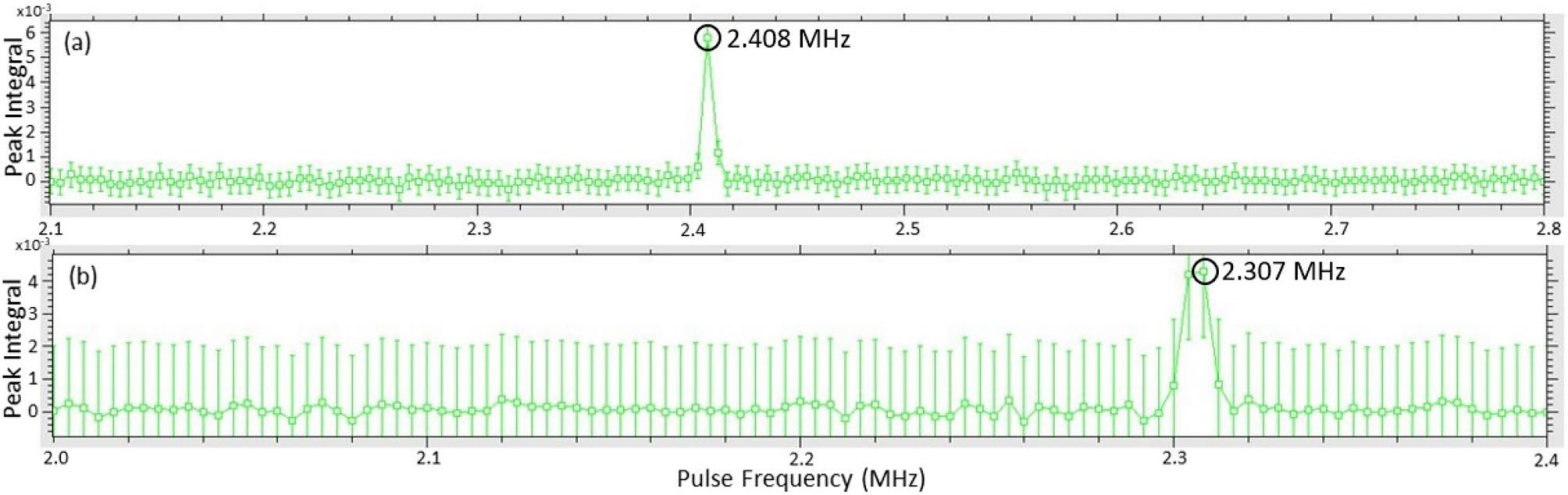
Results of frequency sweeps used to detect $^{14}$N NQR frequencies in the 2–3 MHz range for: (**a**) Benzocaine, and (**b**) Nicotinamide. The detected resonances are labeled using arrows.

**Figure 12:**
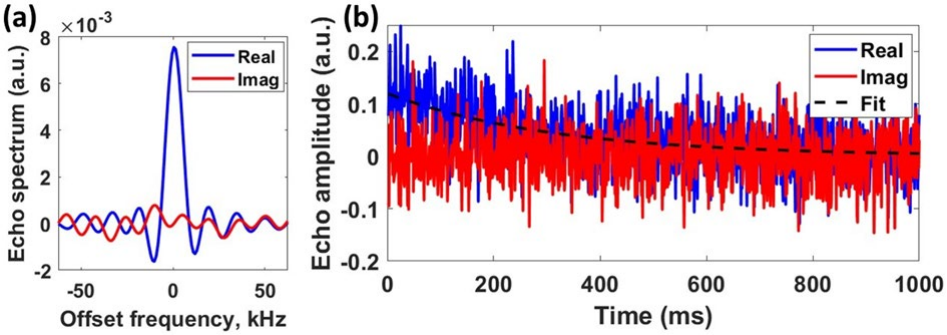
NQR features obtained for a psychoactive drug, namely benzocaine: (**a**) echo amplitude (a.u.), and (**b**) $T_{2,eff}$ (ms).

We performed several experiments on NQR-based detection of both benzocaine and another psychoactive drug, namely nicotinamide. The discovery of NQR resonance frequencies for unknown samples is a prolonged process since it involves searching for a narrow line (typical line width $<10$ kHz) over a broad frequency range ($>2$ MHz) using narrowband excitation (typical bandwidth limited to $<20$ kHz by the tuned impedance matching network). We accordingly performed frequency sweeps over the 1–5 MHz range by varying the excitation frequency, $f_{0}$, in steps of 10 kHz. While this process is slow (typically taking several days), it only has to be performed once for each API. Our measurements yielded newly-discovered $^{14}$N NQR resonant frequencies for both these psychoactive drugs. Specifically, we obtained NQR resonances at 2.408 MHz and 2.307 MHz at room temperature for benzocaine and nicotinamide, respectively, as shown in Fig. 11a,b. We extracted two important features from the raw frequency sweep data. The first is the peak value of the echo spectrum obtained after each scan, which is proportional to the initial echo amplitude *A* and shown in Fig. 12a. The second is the echo decay rate, which is well-approximated by a mono-exponential and thus characterized by an effective transverse decay time constant $T_{2,eff}$ as shown in Fig. 12b.

**Figure 13:**
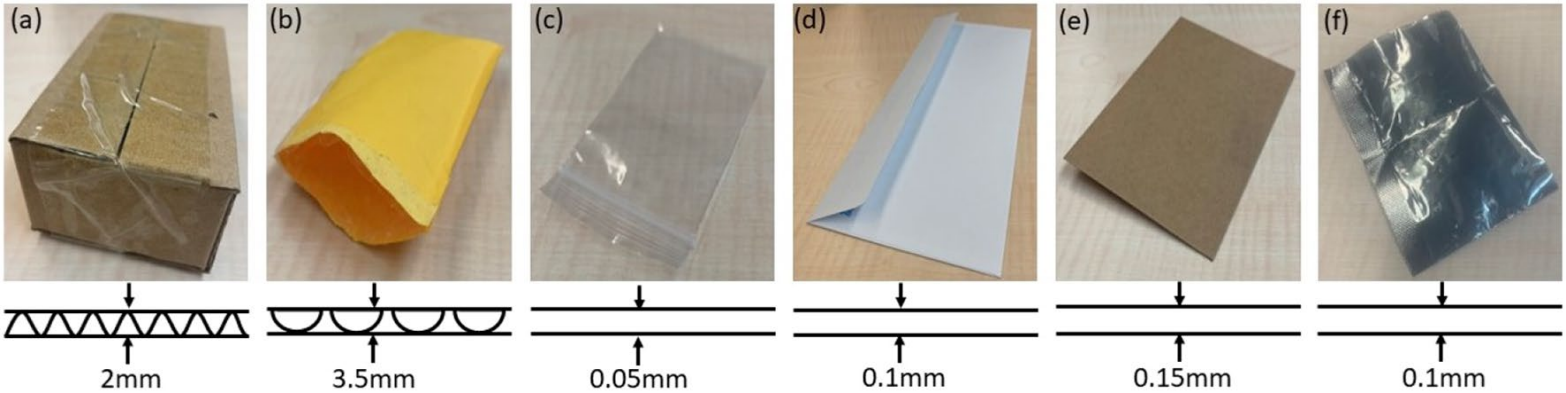
Comparison of different package types investigated in this paper along with their cross-sectional thickness: (**a**) cardboard, (**b**) bubble wrap, (**c**) polyethylene, (**d**) thin paper envelope, (**e**) thick paper envelope, and (**f**) electrostatic discharge (ESD)-protected packaging.

**Figure 14:**
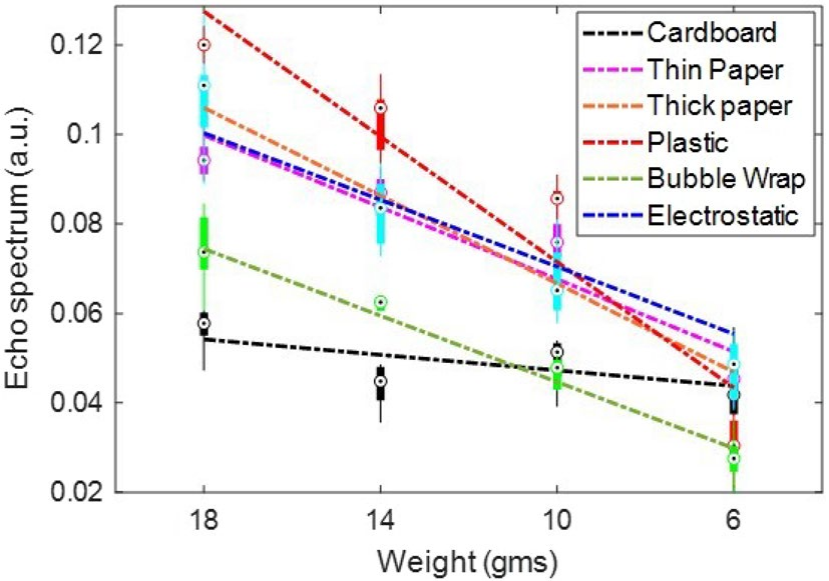
Comparison of $^{14}$N NQR signal amplitudes obtained from the psychoactive drug benzocaine when the sample was enclosed within different types of packaging materials.

**Figure 15:**
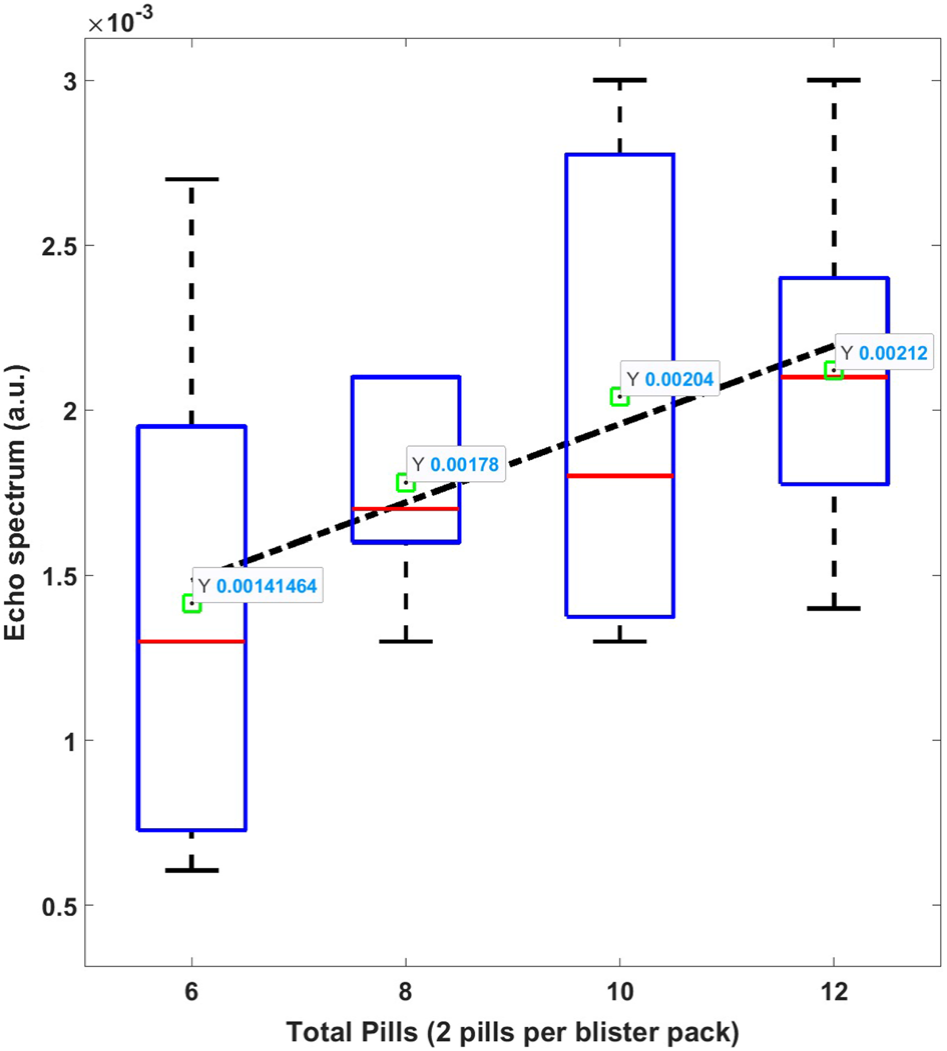
Measured NQR signal amplitudes for acetaminophen pills (325 mg each caplet) packaged within 4 cm × 4 cm blister packs.

### Effect of packaging on NQR signatures

Medicines and drugs come in different kinds of packaging depending on multiple actors, such as manufacturers who use wooden cartons for bulk transport, re-sellers who use cardboard packages, e-commerce vendors who use cardboard, bubble wrap, plastic, and paper envelopes, and pharmacies who use plastic bottles. Most current methods of drug detection require sample preparation (e.g., removal from opaque packaging before carrying out optical spectroscopy). However, it is generally illegal for anyone other than the addressee to open packages. Thus, it is typically not possible to analyze the contents of a mail package if sample preparation is required. This loophole provides willful actors a way to illegally transport restricted medicines and drugs.

To combat this situation, non-invasive analysis techniques are required. Although X-ray or THz scanners can easily penetrate most common types of packaging and are thus non-invasive, they either utilize penetrating radiation, leading to safety issues (for X-rays) and/or require complex (and hence expensive) detection systems. On the other hand, optical techniques such as Raman or IR spectroscopy are safe and chemically specific, but invasive since they cannot penetrate most packaging materials. NQR spectroscopy provides an ideal solution to these problems since it is simultaneously non-invasive, quantitative, insensitive to packaging, sensitive to solid-state chemistry and crystal structure, inherently safe, and realizable using portable and low-cost instrumentation (Fig. 13).

We experimentally validated the advantages of NQR for this application by placing benzocaine samples within different kinds of thin packages, including cardboard, bubble wrap, paper envelopes, plastic envelopes, blister packages, and electrostatic discharge (ESD)-protected mailers (as shown in Fig. 14) and then measuring the resulting NQR spectra. The goal was to study the efficiency of NQR in identifying the contents of the package in each case. The maximum amount of benzocaine for which the linear approximation in Fig. 10 remains valid was found to be $\sim$18 g across different packages.

The main effect of non-conductive packaging materials on the NQR signal simply results from a physical shift in the position of the sample with respect to the surface of the planar coil. This shift arises from the thickness of the packaging material and is obviously larger for thicker packages. Since the $B_1$ field weakens with depth, *z*, the effect is to decrease the measured NQR signal amplitude as predicted by Eq. (2). This trend can be clearly observed in the experimental NQR data from different packages shown in Fig. 14. Relatively thin packages such as paper and plastic result in the largest signal amplitudes, while thick packages such as cardboard and bubble wrap result in the smallest amplitudes. The dependence of signal amplitude on package type can be removed if desired (e.g., in order to quantify the amount of API within the package) by combining calibration data of the type shown in Fig. 14 with a visually-derived estimate of package type (or thickness). An example is described in the next sub-section.

To evaluate the effects of conductive packaging, we also conducted experiments with acetaminophen pills placed inside blister packages with aluminum foil backing layers. Figure 15 plots the resulting NQR signal amplitudes versus the total number of pills, each of which contained 325 mg of the API. In general, blister packs result in lower NQR signal amplitudes (and thus SNR) than other packaging methods. The main reason is that samples in blister packs are separated into small pills or capsules, resulting in lower API concentrations than for bulk samples. Some ways to deal with this challenge include (1) redesigning the coil to fit tightly around the blister pack, thus increasing its detection efficiency; and (2) using other techniques for enhancing signal amplitude, such as sample prepolarization^31^. Note that for conductive packages such as most blister packs, the NQR signal amplitude is further reduced by an additional effect, namely decreased $B_1$ field strength at a given depth due to eddy currents.

### API quantification

Consider the problem of non-invasively estimating the amount of a known API (e.g., benzocaine) given basic information on packaging type. The data in Fig. 14 suggests that a linear model for the relationship between API weight and NQR signal amplitude should be adequate over the range of weights studied (6-18 g). In general, such a relationship can be written as

3 $$\begin{aligned} q = \frac{A-c}{m},\quad q_{max}\ge q\ge q_{min} \end{aligned}$$

where *q* is the weight of the sample under test, $[q_{min},q_{max}]$ is the range of weights for which the linear model is valid, and *m* and *c* are the model parameters (slope and offset). The best-fitting values of *m* and *c* for different package types are summarized in Table 2.

**Table 2:** Constants used in Eq. (3) for estimating the quantity of benzocaine in different packages.

Package type	Slope (*m*)	Constant (*c*)
Cardboard	0.00086	0.0386
Thin paper	0.0040	0.0273
Thick paper	0.0049	0.0174
Plastic	0.0070	0.0013
Bubble wrap	0.0037	0.0076
Electrostatic	0.0037	0.0330

## Discussion

**Figure 16:**
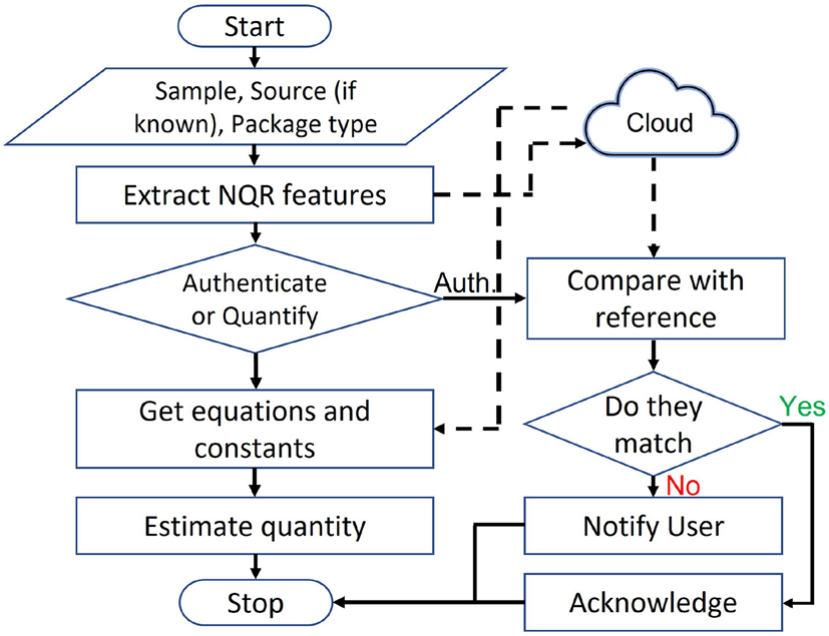
Proposed model for authentication and quantification of mail packages using NQR spectroscopy.

Our results show that NQR can be used to detect and quantify psychoactive drugs non-invasively. NQR-based authentication models can therefore be integrated into different stages of the mail life cycle to ensure the security of the packages from counterfeiting and adulteration. A sample authentication model is shown in Fig. 16. According to the proposed model, the system can be used to either non-invasively authenticate a package or quantify the amount of API it contains. The system is connected to the cloud to obtain updated information on different samples, their reference NQR feature vectors (also known as signatures), and other knowledge required to estimate the quantity of API. In the case of authentication, the user is notified if there is any discrepancy between the expected and measured signal properties.

Unknown NQR resonance frequencies (e.g., of new psychoactive drugs) can be found by either (1) carrying out frequency sweep experiments over the ranges suggested, (2) analyzing existing data from similar compounds, or (3) performing first-principles density functional theory (DFT) calculations to estimate the electric field gradient tensor surrounding the NQR-sensitive nucleus being studied^33^. The acquired knowledge can then be used to detect drugs being transported through the mail. Since there are a variety of drugs in the market, the availability of a broadband NQR system^34^ would enable straightforward control over the transmitted and received frequencies and thus strengthen the proposed authentication model. In future work, we also plan to extend our authentication approach to a cloud-based model that automatically updates in-field systems with new (or updated) NQR feature vectors.

## Conclusion

This paper has presented a novel model for non-invasively authenticating mail packages using nuclear quadrupole resonance (NQR) spectroscopy. In most previous studies, the use of $^{14}$N NQR for detecting small quantities was overlooked since it has relatively low intrinsic sensitivity. This drawback is much less of a concern when authenticating large sample sizes, such as those being routinely transported through the mail system. Thus, we developed and presented a system for NQR-based authentication of mail packages that can reliably authenticate packages and/or quantify its contents given only basic knowledge, such as the type of packaging material. We believe that the widespread adoption of such systems has the potential to significantly reduce the illegal transfer of controlled substances across national borders.

## Data Availability

The datasets generated and/or analyzed during the current study are available in the *Raw-Data* repository located at https://github.com/khoraceherron/Raw-Data.git.
